# Supplementary material for: Patterns of Intron Gain and Loss in Fungi
Source: PLoS Biol. 2004 Nov 30;2(12):e422. doi: 10.1371/journal.pbio.0020422 (PMC532390; doi:10.1371/journal.pbio.0020422)
Supplement: Table S1 — Also available at http://genes.mit.edu/NielsenEtAl/. (4.3 MB ZIP). [file pbio.0020422.st001.zip › NielsenEtAl/html/779.html]

AN6567.1.NCU03834.1.MG06932.1.FG07405.1


```
 CLUSTAL W (1.82) Multiple Sequence Alignments - Introns Inserted


Sequence 1: NCU03834.1	589 aa
Sequence 2: MG06932.1	578 aa
Sequence 3: FG07405.1	644 aa
Sequence 4: AN6567.1	570 aa
Alignment Length: 668 aa
Number Identitical Residues: 159 aa
Alignment Score (without introns) 9215


MG06932.1 	----MATRKVRYKKLNVKTPLPVLLFSEIDSNEYEALKNE-QIQTGVESAEEN0EYHLQA
NCU03834.1	----MATRKVRYKKLSVKTQLAVLREDQIEASEYESLTSENQIATGVEQAEEN0EYHLQA
FG07405.1 	----MSSRKVRVKKLNVKTTLPVLREDQIDPNEYEALTTDNQIATGVEQAEEN0EYHLQT
AN6567.1  	MTRYAGLGRTRPKKLTPKAPIPIYREHQIDDLEEEIQNGLQQVETGVEKAEES0EYHLQV
          	 :   .  :.* ***. *: :.:    :*:  * *  .  .*: ****.***. *****.

MG06932.1 	VLSTTG---VAADKEIPVTQTQESNINYDELYPTKFVEPTNYIRFSQTVEECIGCPYDMT
NCU03834.1	VLKGAG---VAADQEIPVPPPQQSELDYDQFYPQKVAKTSTYIRFSQTVEECISCLYDMT
FG07405.1 	ILKEAG---TSNDQEIPVPPPQESDINYDDLYPVPFHKPSSYIRFSQTVEECITCLYDMT
AN6567.1  	AINAVASGRVVNEAHIPTPETVLSNLQYDELYPPVFSQPATYIRFSSTVEDCCGCPYNMT
          	 :. ..:. .  : .**.. .  *:::**::**  . :.:.*****.***:*  * *:**

MG06932.1 	EDDEAFLAKYNKDLAAGTAALSEDDFEKIMFVFEDTASVQAPYAAVDNT-VVSYDHMVPP
NCU03834.1	EDDETFLKSYNMKLTP-SARLSEDDFERIMDVYEDMAANITPFSAIDQT-VPSYQEMLRG
FG07405.1 	TEDDEFLKQYNSKPPA-TGVLSEDDFEHIMEVFEDTAAEQTPFAAVDNT-VAAYDMMLPG
AN6567.1  	DEDDVFLKIMNEKRDP-ADRCTEDQFEEVMNFFEETVRLKQPYAAVGSAPVLSFAEMQES
          	 :*: **   * .  . :   :**:**.:* .:*: .    *::*:..:.* ::  *   

MG06932.1 	LTELEL---PRLMLHAKAIYEHWKSQRQAKKG-PLHPSLK~FETHQESDDLDPFVCFRRR
NCU03834.1	LEPLDS---TKVMVHAKQIYEYWKSRREISKNRPLNPTLK~FETHAESDELDPYVCFRRR
FG07405.1 	LTHLNQSVSTDVLQHAKPVYEYWKSRRQEAGNKPLHPSLK~FETHQETDDTDPFVCFRRR
AN6567.1  	MDATVE---DYVRRLAKDVYDHWKTRRLNNGNQSLLPSLK0FETGAETDDTDPYVCFRRR
          	:          :   ** :*::**::*    . .* *:** ***  *:*: **:******

MG06932.1 	EVRQTRKTRARDTQIADKLKRLRRELEEGRELIALAHGREVMKREMLNVDRAIFEQRCNV
NCU03834.1	EIRQTRKTRARDVQSADKLKRLRKELEEGRQLILAAHNRELLKADMLKVERAIFDQRAII
FG07405.1 	EARQTRKTRARDNKIAETLKKLRRELEDGRQLVLLAYEREMVKREVMSMDRAIFEERARL
AN6567.1  	EVRQVRKTRGRDAQSADKLRRLRKELEDARQLVALVRQRELARKEMLATERILFLQRAEV
          	* **.****.** : *:.*::**:***:.*:*:  .  **: : :::  :* :* :*. :

MG06932.1 	KKVKIKLGIKTDDEDLYNTK0PQRKKPAEAQPVQQRAAAGPARVAPRSDTRASDVDLGLL
NCU03834.1	KEQKLRLGIRTGDEDLVNQK0PQKRKAPEAPSAQRPPPPPQIRMPVRPDGRPAESDLVQL
FG07405.1 	KDMKLRLGIKGEDEDLVNQK0PQKRKPVEPPVVRQPTG-AHLRQPVRSDGRTLDADLVLL
AN6567.1  	KDMKRKLNIKDDDEDLINQK0P-KKKPIEAPPMQRPAA-AQLRMPPKP-GAQAAEDLQLL
          	*. * :*.*:  **** * * * ::*. *.   :: .     * . :.       **  *

MG06932.1 	SELKAQREEEMRVDIETKIENHKKWNEKYVDLTNSSLVDKKPETEQEPDPAKWRPVKVQY
NCU03834.1	SDRLAEKNAELIIEIEKKIQNHIDWNKNYVDLTGKPLS---PVQGPRQD-LGFRPAKTQY
FG07405.1 	SDKLAEKETELRLDIEMKVQNHRKWNQNHIDLTREPLS---PVKEQGTE-VKFRQAKTQY
AN6567.1  	EDVQAEKENEIIRDIKANIAKHMKWNEGYVDFTRAPLS---PSPERTVD-ISFRPAITTQ
          	.:  *::: *:  :*: :: :* .**: ::*:*  .*    *      :   :* . .  

MG06932.1 	LMTPPNSASAE-DLEEPTP--MELDAPAPAPVR--RFHGLSADDEDYSPDPMEYRRRVGR
NCU03834.1	LMTPPASASSG-SMDEPTP--MDLDKPKPNPPPPVKFRGVAQD-EQSLAHPPSYRRRIGR
FG07405.1 	LMTPPASTSSEMEVDEIAPDAMQLDKRES-----SVFQFTAGSGEQSKGSQPAFRRRIGR
AN6567.1  	LPTPPSSDSSE-----NTP-DLALDMSGTVSYR-DKLDEHALIMSEDANKMPSFRRRIGR
          	* *** * *:       :*  : **   . .     :   :   .:       :***:**

MG06932.1 	LNRLWVDRRPVKKQLVDVVPTRFPVVSDRWKYDCDSSDDEDS-VYEVDPFDLRALKFRAT
NCU03834.1	LNRLWIDRRGLPSPARDLSEE----QSDRWKYDQSSDDEDDAPVYMLDPFDTKALRYRAS
FG07405.1 	LNRLWIDRRGMVTPPRELGED----RSDRWKYDSDSDDEEPP-VYEVDPFDTRALKFRAT
AN6567.1  	GGRLLIDRRNFASRCRVELDP---WKADRFKYDQEDSDEDLD--YEMDQYDISLMQNRAI
          	 .** :*** . .             :**:*** ...*::    * :* :*   :: ** 

MG06932.1 	IPLGSAAP-------RRIEGSAAAP-----VTNGTARPALPWSGGGQPPSQ-------QP
NCU03834.1	IPLQTVTRPPPPVINRQFIPPGAVPQ---QLAQSSFAPGQPQPQSQPQPNQSQSLPLPQP
FG07405.1 	IPLNPYMFRGRPAVPPEAVAAAQAQAGNRVLPSPAAAAAHAHAQAHAQAAAAAHLAAQAQ
AN6567.1  	MLAKARDQ-------AHAQAQAAQ------VRRLQAEQAALNNLNSGQTSG---------
          	:   .           .    .        :       .         .           

MG06932.1 	QQQQQQPQPQPQLQMQ~QSA----------------------------------------
NCU03834.1	QQPVAQPQPQPQPQAQ~PVS----------------------------------------
FG07405.1 	AKAQAVAQQQAQAAQE1QSGGPLGKVDKPGGHITRRVYYLVADEGIARSFGAGGRFSGLN
AN6567.1  	QTMGSNPGPGAIAPTP~ET-----------------------------------------
          	      .   .                                                 

MG06932.1 	------------
NCU03834.1	------------
FG07405.1 	VGQIACLMARVE
AN6567.1  	------------
          	
```
